# Real-time measurements of vascular permeability in the mouse eye using vitreous fluorophotometry

**DOI:** 10.1038/s41598-023-36202-4

**Published:** 2023-06-07

**Authors:** Nadine Colé, Janina Thoele, Christoph Ullmer, Richard H. Foxton

**Affiliations:** Ophthalmology Discovery, Pharma Research and Early Development, Roche Innovation Center Basel, F. Hoffmann-La Roche Ltd, Basel, Switzerland

**Keywords:** Biomarkers, Pharmacology, Target validation, Biological models, Neuro-vascular interactions, Retina

## Abstract

Breakdown of blood-retinal barrier integrity underpins pathological changes in numerous ocular diseases, including neovascular age-related macular degeneration (nAMD) and diabetic macular edema (DME). Whilst anti-vascular endothelial growth factor (VEGF) therapies have revolutionised disease treatment, novel therapies are still required to meet patients' unmet needs. To help develop new treatments, robust methods are needed to measure changes in vascular permeability in ocular tissues in animal models. We present here a method for detecting vascular permeability using fluorophotometry, which enables real-time measurements of fluorescent dye accumulation in different compartments of the mouse eye. We applied this method in several mouse models with different increased vascular leakage, including models of uveitis, diabetic retinopathy and choroidal neovascularization (CNV). Furthermore, in the JR5558 mouse model of CNV, we observed with anti-VEGF post-treatment a longitudinal reduction in permeability, in the same animal eyes. We conclude fluorophotometry is a useful method for measuring vascular permeability in the mouse eye, and can be used over multiple time points, without the need to sacrifice the animal. This method has the potential to be used in both basic research for studying the progression and factors underlying disease, but also for drug discovery and development of novel therapeutics.

## Introduction

Loss of blood-barrier integrity leading to oedema and subsequent tissue damage has been described as a contributory factor in a wide variety of diseases, including sepsis^1^, cancer^2^, and stroke^3^. In the eye, vascular instability and buildup of fluid within the retina and surrounding tissues is a central feature of some of the most common-sight threatening diseases, such as neovascular age-related macular degeneration (nAMD) and diabetic macular oedema (DME), which if left untreated can lead to rapid vision loss^4–7^. Furthermore, ocular diseases such as glaucoma^8^ and uveitis^9^ have also been linked to increased vascular permeability, although further research may be required to define this link to the disease etiology.

Intravitreally injected anti-VEGF inhibitors, such as ranibizumab and aflibercept have become a standard of care for treating nAMD and DME^10,11^, and have revolutionised patient visual outcomes, although these diseases continue to be leading causes of visual impairment^12^, as not all patients respond to anti-VEGF treatment, and some patients may continue to experience retinal or subretinal fluid leakage, or develop fibrosis and atrophy despite treatment. To help address one of these unmet needs the next generation of therapeutics have recently entered the market, aiming to restore vascular stability in the eye, such as faricimab (VABYSMO; Genentech/F. Hoffmann-La Roche Ltd.), a vascular endothelial growth factor (VEGF)–angiopoietin (Ang)-2 bispecific antibody^13–15^.

Development of novel treatments that are able to prevent pathological ocular vascular leakage continues, as our understanding of these diseases evolves. To help develop these new therapies, preclinical models and respective methods are required that allow changes in vascular permeability to be monitored over time. Current permeability methods include Evan’s blue^16,17^, Fluorescein isothiocyanate (FITC)-dextran^18^ and microsphere^19^ perfusion techniques, as well as imaging-based techniques such as fluorescein angiography^20,21^, and exogenous contrast-enhanced leakage optical coherence tomography (OCT)^22^. Whilst many of these techniques are well-established, they may also have some challenges and drawbacks, which are discussed in greater depth elsewhere^20^. The method described here may also be used in conjunction with existing techniques.

Fluorophotometry is a versatile tool that has long been used in both clinical and nonclinical ocular research settings^23,24^. Fluorophotometer instruments have been designed to measure the concentration of fluorophores within the ocular tissues^25^, and thus can be used to determine the diffusion and elimination of the reference dye, to provide an indication of the physiological status of the ocular vasculature. Fluorophotometry instruments have been available for ocular applications such as aqueous flow, drug pharmacokinetics, tear film production, and vascular leakage in human^26^, rats^27,28^ and other species^29,30^, but until relatively recently^31^ it was not possible in mice, due to the limitations of the size of the eye. In this paper we examine the use of a novel accessible technology for measuring permeability in the retina, vitreous and anterior chamber. We attempt to validate the technology in animal models of ocular disease, as well as providing an example of how it could be used to measure vitreous permeability following anti-VEGF treatment, to demonstrate its potential in developing therapeutics. These findings indicate that fluorophotometry may be utilised to monitor and quantify changes in vascular leakage in the mouse eye, which could also be used alongside other existing methods.

## Results

**Figure 1:**
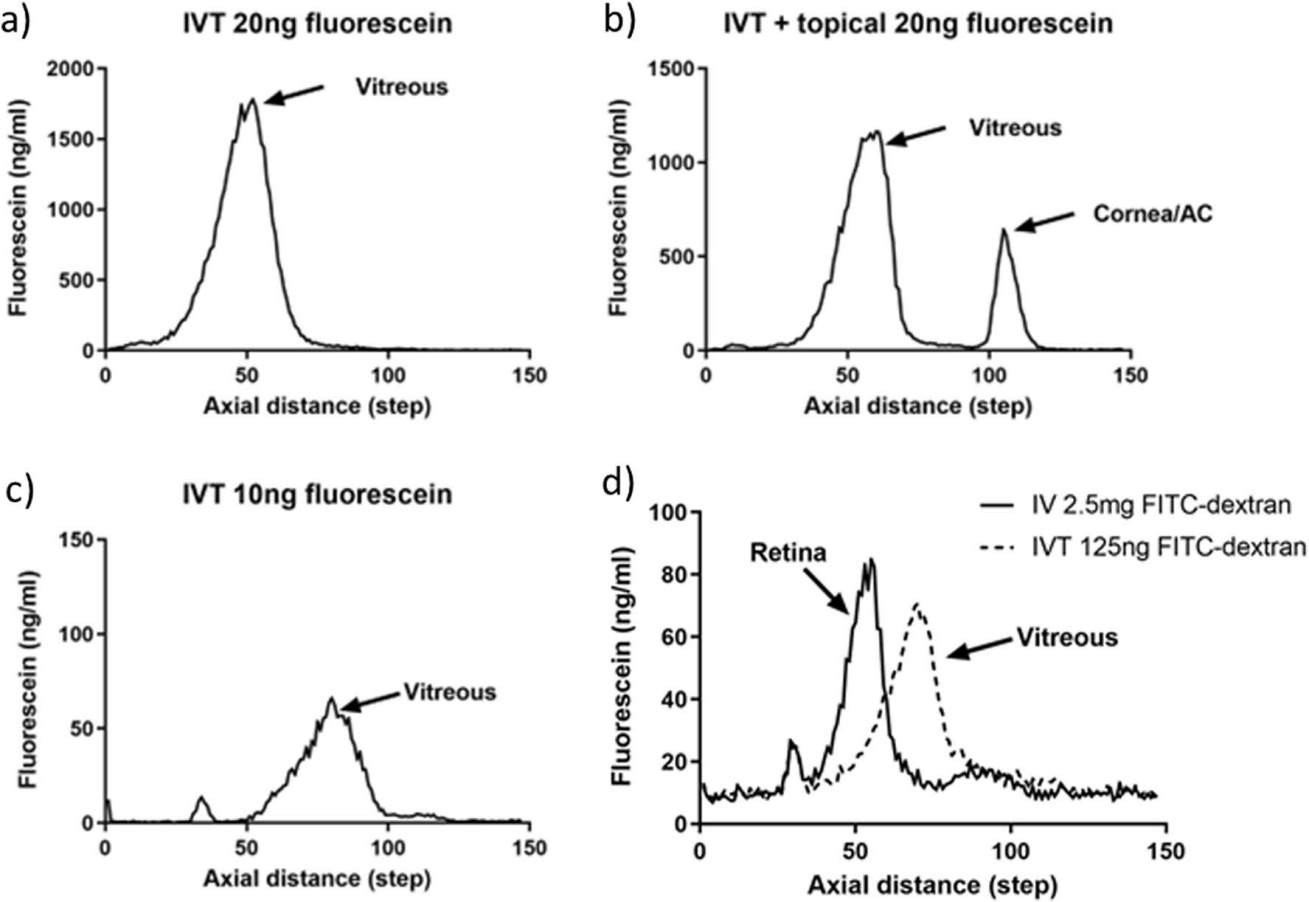
Differentiating the different compartments of the eye on scans from the mouse fluorophotometer. Fluorophotometer scans performed on C57Bl/6J mouse eyes, following; (**a**) intravitreal injection of 20 ng of fluorescein, (**b**) intravitreal injection of 20 ng of fluorescein, followed by 20 ng of fluorescein applied topically, (**c**) intravitreal injection of 10 ng of fluorescein, and (**d**) intravitreal injection 125 ng of 70 kDa FITC-dextran and intravenous injection of 2.5 mg of 70 kDa FITC-dextran (scans carried out in separate mice and combined on the same plot). Intravitreal injections were performed into the central vitreous. Labels on each Figure show the location of the vitreous (central), anterior chamber and retina for each mouse eye. AC = anterior chamber; IVT = intravitreal.

The initial step to validate the use of the mouse fluorophotometer, was to allocate the axial distances of the scan corresponding to the retina, vitreous, and cornea/anterior chamber of the mouse eye. To detect the vitreous, we began by injecting 20 ng fluorescein directly into the central vitreal space, followed by immediate measurements with the fluorophotometer through the central vitreous. A sharp peak was observed between 69 and 77 axial steps (Fig. 1a). Following this, we then administered 20 ng of fluorescein topically to the same mouse eye, to show the position of the cornea/anterior chamber. A peak between 111 and 116 axial steps was identified for the cornea/anterior chamber in addition to the vitreous peak (Fig. 1b). In a second mouse, intravitreal (IVT) injection of 10 ng of fluorescein (Fig. 1c) resulted in a vitreous peak of approximately half the size of the 20 ng dose (Fig. 1b), but on the same axial distance. This demonstrated reproducibility of the axial distance independent of the fluorescein dose, and a dose-dependent peak size of the IVT injection. However, we were unable to separate cornea and anterior chamber measurements, due to spatial resolution constraints.

Following identification of the vitreous and cornea/anterior chamber, we then sought to differentiate vitreous and retinal compartments. Using 70 kDa FITC-dextran, whose large size compared to fluorescein (molecular weight = 376 Da) prevents it from penetrating the blood-retinal barrier and diffusing from the retina into vitreous, we injected one mouse intravenously (IV) with 2.5 mg FITC-dextran, and compared peaks with another mouse given 125 ng FITC-dextran IVT. In the IV infused mouse, a peak was detected at 51–55 axial distance, corresponding to FITC-dextran accumulation in the retina, whereas in the IVT administered mouse eye the vitreous peak was detected at 69–74 axial distance. Overlay of both traces shows that separation of the retinal and vitreous compartments is possible (Fig. 1d). The path lengths for retina, vitreous, and cornea/anterior chamber identified in Fig. 1 served as a reference for future eye measurements.

**Figure 2:**
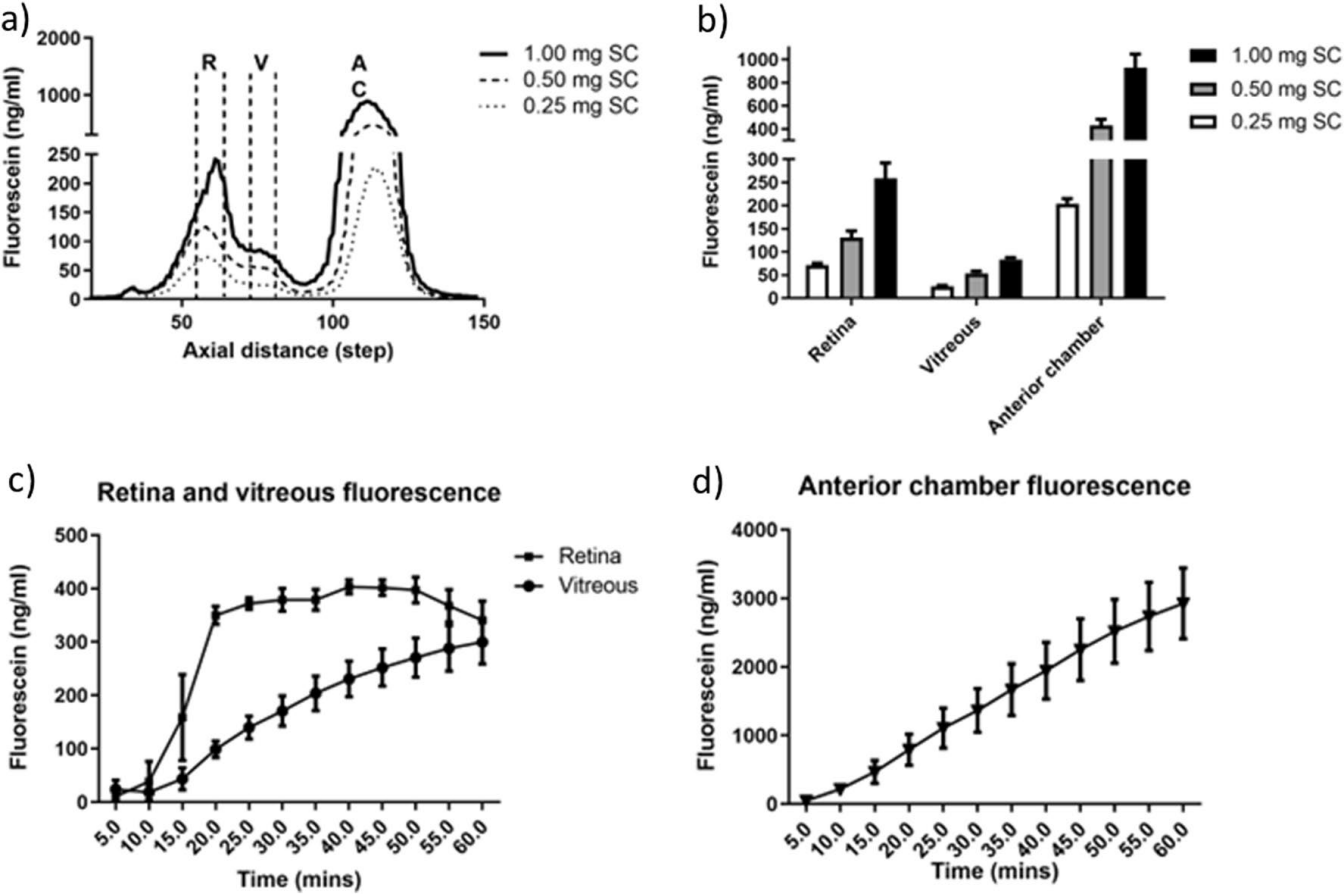
Dose–response and time courses following subcutaneous administration of fluorescein to female C57Bl/6J mice. (**a**) Representative scans and (**b**) quantification of fluorescein in the retina, vitreous and anterior chamber averaged after 30 min, from mice injected with 0.25, 0.50 and 1.0 mg of fluorescein subcutaneously. Time courses showing fluorescence levels in (**c**) the retina and vitreous, and (**d**) the anterior chamber of C57Bl/6J mice, 5–60 min after subcutaneous injection of 1 mg of fluorescein. N = 5–6 mice for dose-responses in (**a** and **b**), and N = 3 mice for time courses (**c** and **d**). R = retina; V = vitreous humour; AC = anterior chamber; SC = subcutaneous.

After identification of the different mouse eye compartments by fluorophotometry, our next goal was to determine the sensitivity of the fluorophotometer instrument to detect changes in dose levels of fluorescein in the eye. We decided to use systemically administered fluorescein, since this has been the fluorophore of choice for multiple previous studies^31–33^, which we could then compare with. We chose initially to test two systemic routes for fluorescein injection, IV and subcutaneous (SC). IV dosing has the advantage that it allows direct administration of the dye into the circulation, so does not introduce additional adsorption variables. However, in our hands the accurate injection of small volumes lead to higher variability, and more mice may be required to generate datasets (Supplemental Figure 1). In contrast, SC dosing (Fig. 2) produced robust changes in fluorescence intensity, with low inter-animal eye measurement variability.

In an initial experiment we used increasing doses of 0.25, 0.5 and 1.0 mg of fluorescein injected SC, and after 30 min performed scans of the eye. We observed a dose-dependent increase of fluorescein in each compartment as shown in the average scan profile (Fig. 2a), which could be quantified by area under the curve (AUC) measurements of the fluorescent signals in the retina, vitreous and anterior chamber (Fig. 2b). From this experiment we selected the 1 mg dose for time course experiments shown in Fig. 2c, d, as this dose was optimal for detection of fluorescence in the different mouse eye compartments.

We next monitored the accumulation of fluorescein in the different eye compartments every 5 min, up to 60 min post-injection. In the vitreous (Fig. 2c) and anterior chamber (Fig. 2d), a linear elevation in fluorescein was detected. In the retina, fluorescein levels saturated around 20 min after SC injection (Fig. 2c). With IV dosing (Supplemental Fig. 1), similar trends were observed compared to SC dosing in the anterior chamber and vitreous, however retina levels of fluorescein peaked at 5 min after administration, and reduced over the course of the experiment presumably due to higher systemic clearance.

**Figure 3:**
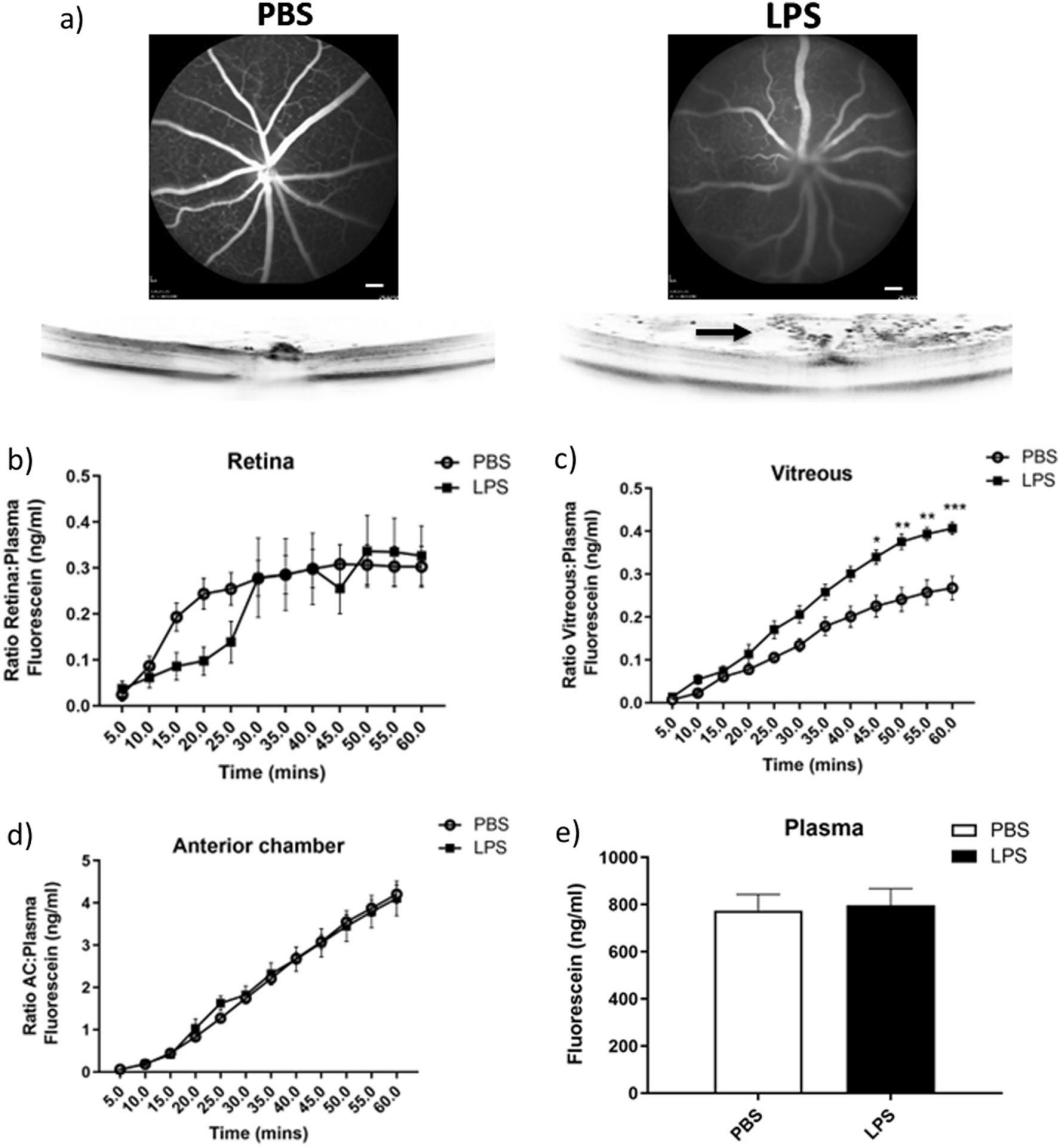
Application of the mouse fluorophotometer in the endotoxin-induced uveitis model. LPS (1 ng) or PBS was injected intravitreally into C57Bl/6J mice eyes, and 24 h later, fluorophotometry, OCT and fluorescein angiography were performed from mice injected with 50 mg/kg fluorescein subcutaneously. (**a**) Representative fluorescein angiography (upper panels) and OCT images (lower panels), from PBS (left) and LPS (right) treated mouse eyes 50 min after fluorescein administration. Arrow in the OCT image from the LPS-injected eye shows the presence of infiltrating immune cells in the vitreous. (**b**–**d**) Time courses quantifying levels of fluorescein in (**b**) the retina, (**c**) the vitreous, and (**d**) the anterior chamber using fluorophotometry revealed no significant differences in fluorescein levels in the retina and anterior chamber between PBS and LPS treatment. However, in the vitreous, a significant increase at the later time points from 45 to 60 min was observed following LPS treatment. Data shown in (**b**–**d**) are corrected to plasma levels of fluorescein. (**e**) Plasma levels of fluorescein were not significantly different between PBS and LPS treatment groups. Scale bars = 500 µm. *N* = 5–8 eyes per time point. *P < 0.05, **P < 0.01, ***P < 0.001, using Two-way ANOVA with repeated measures, followed by Bonferroni’s multiple comparisons. Data are given as means ± SEM. PBS = phosphate buffered saline; LPS = lipopolysaccharide.

**Figure 4:**
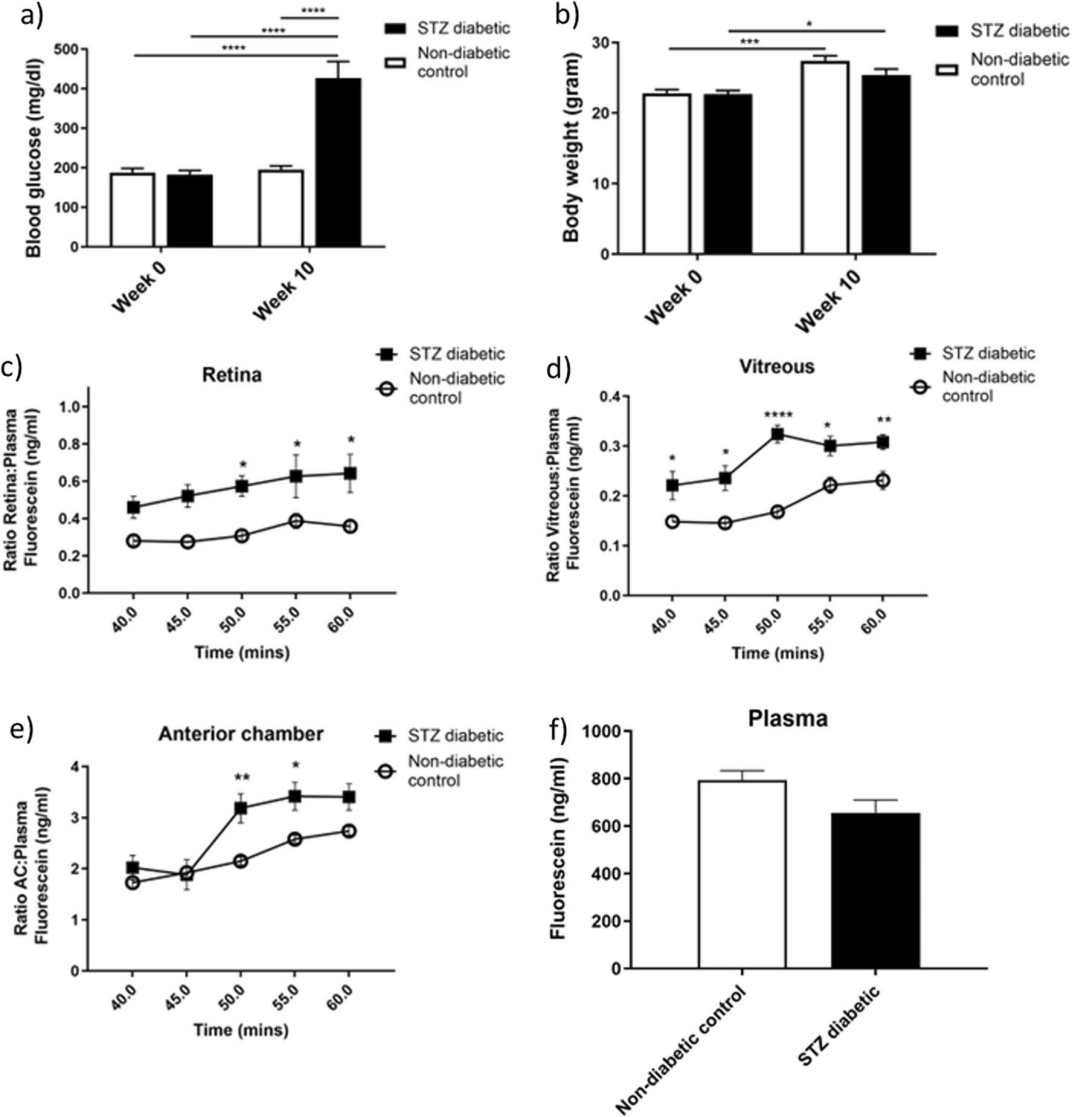
Quantification of permeability in the streptozotocin (STZ) diabetic mouse model using fluorophotometry. Mice that had been induced with STZ to be diabetic 10 weeks previously were used in this experiment, and compared with age-matched non-diabetic control animals. (**a**) Blood glucose levels were significantly elevated in STZ diabetic mice, 10 weeks following diabetes induction, whereas (**b**) body weight was not significantly different from non-diabetic control mice. Levels of fluorescein in (**c**) the retina, (**d**) the vitreous and (**e**) the anterior chamber were all significantly increased in STZ mice at various time points from 40 to 60 min post-injection of 50 mg/kg subcutaneous fluorescein, compared with non-diabetic controls. Data shown in (**c**–**e**) are corrected to plasma levels of fluorescein. (**f**) Plasma levels of fluorescein were not significantly different between STZ diabetic and non-diabetic control mice treatment groups. *N* = 6–16 eyes per time point. Blood glucose and body weight assessed statistically by Two-way ANOVA with repeated measures, followed by Tukey’s multiple comparison test. Ocular compartment leakage assessed statistically by Two-way ANOVA with repeated measures, followed by Bonferroni’s multiple comparison test. * P < 0.05, ** *P* < 0.01, **** *P* < 0.0001. Data are given as means ± SEM.

**Figure 5:**
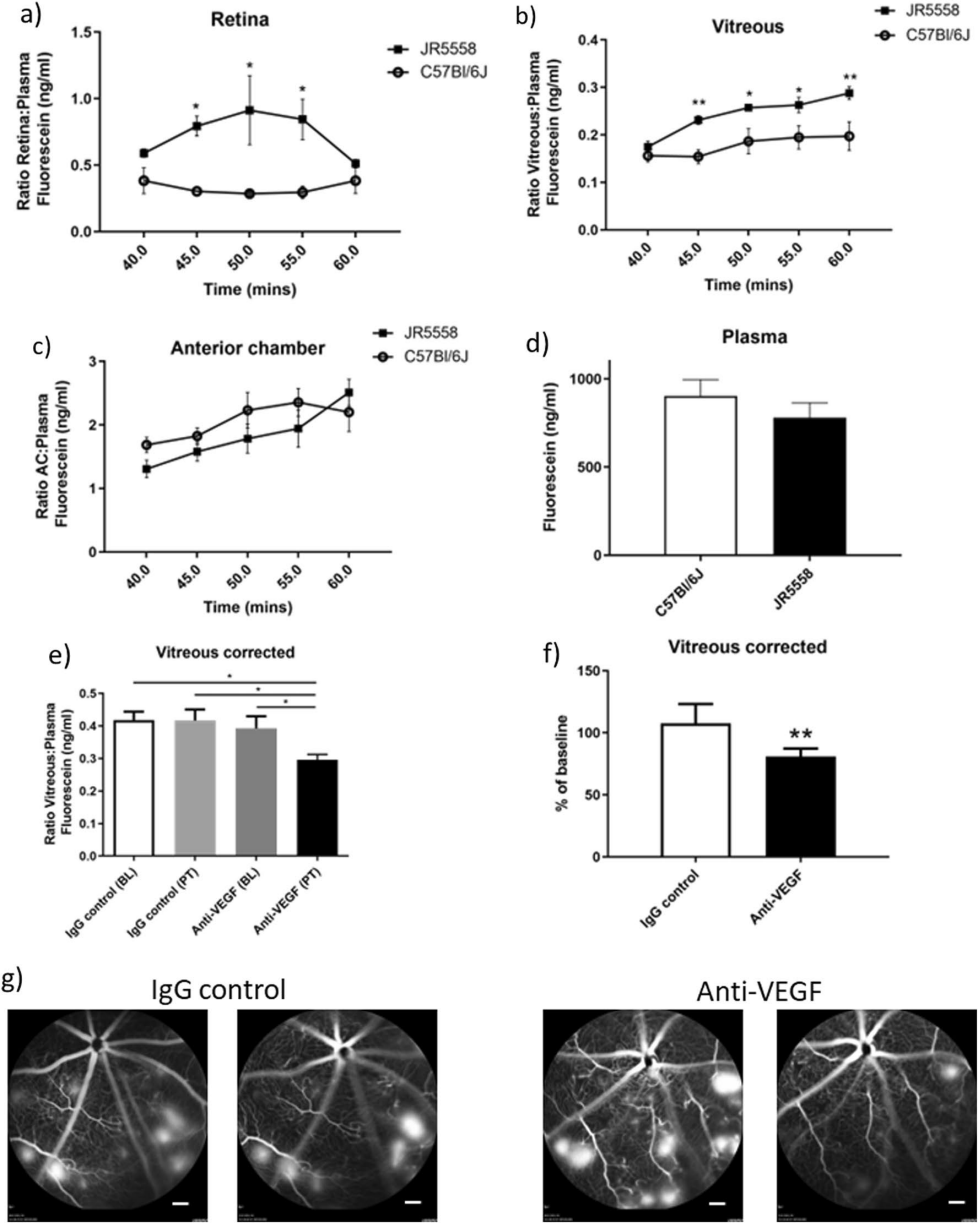
Quantification of permeability in the JR5558 spontaneous CNV mouse model, and response to anti-VEGF treatment measured using fluorophotometry. (**a**–**c**) Male and female JR5558 and age-matched control C57Bl/6J mice were injected subcutaneously with 50 mg/kg fluorescein, and time courses of ratios of fluorescein levels corrected to plasma in separate eye compartments were measured. JR5558 mice had significantly increased fluorescein levels in (**a**) the retina and (**b**) the vitreous compared with C57Bl/6J mice. Levels of fluorescein in (**c**) the anterior chamber and (**d**) plasma were not significantly different. (**e**–**g**) JR5558 mice were dosed at baseline (day 0) and day 3 intraperitoneally with anti-VEGF or IgG control antibodies, and fluorescein leakage was quantified using fluorophotometry at baseline, and day 7. Levels of fluorescein corrected to circulating plasma were measured 45 min after subcutaneous fluorescein administration. (**e**) Anti-VEGF treatment significantly reduced the amount of fluorescein in the vitreous to approximately 77% of baseline (**f**), whereas IgG control had no significant effect. (**g**) Representative fluorescein angiography images of JR5558 mice eyes at baseline (left panels for each treatment group) and post-treatment (right panels for each treatment group) following IgG or anti-VEGF treatment. *N* = 3–6 eyes for time courses, *N* = 12 eyes per group for IgG and anti-VEGF treatments Scale bars = 500 µm. Ocular compartment leakage assessed statistically by Two-way ANOVA with repeated measures, followed by Bonferroni’s multiple comparison test. Baseline and post-treatment corrected vitreous fluorescein concentrations were assessed by One-way ANOVA followed by Newman Keul’s multiple comparisons test. Percentage change from baseline assessed statistically by unpaired T-test. *P < 0.05, **P < 0.01. Data are given as means ± SEM.

As a result from these experiments, we decided to continue with a dose of 50 mg/kg fluorescein, for all subsequent experiments as shown in Figs. 3, 4, and 5. The 50 mg/kg dose approximates to a 1 mg total fluorescein dose used for previous time course experiments for a mouse of around 20 g. Adjustment to body weight ensures more consistent fluorescein plasma levels. In addition, for the remaining experiments, levels of fluorescein in the different eye compartments were normalised to plasma values, and expressed as such.

After selection of the dose and route of administration of fluorescein, we began to test the suitability of the mouse fluorophotometer for detecting vascular permeability changes in disease models. The first we investigated was endotoxin-induced uveitis (EIU), a model of anterior uveitis. The endotoxin LPS was injected IVT, to induce inflammation and blood-retinal-barrier breakdown, and then fluorescein angiography, OCT and fluorophotometry were carried out 24 h later (Fig. 3). Treatment of eyes with LPS induced vascular leakage as observed via fluorescein fundus angiography, with a concomitant increase in inflammatory cell influx, which could be observed by OCT imaging (Fig. 3a). In parallel, we carried out fluorophotometry, and in animals treated with LPS there appeared to be slower fluorescein uptake between 15 and 25 min in retina when compared to PBS injected mice (Fig. 3b), however this did not reach significance. Furthermore, at 30 min and later there were no significant differences in fluorescein levels compared to the PBS control group, as was also the case for the anterior chamber (Fig. 3d). In contrast, in the vitreous compartment, we observed higher levels of fluorescein in LPS treated eyes, which reached significance after 45 min and continued to 60 min (*P *< 0.05; Fig. 3c). Levels of plasma after 60 min were not significantly different between LPS and PBS groups (Fig. 3e), thus differences in vitreous levels of fluorescein were likely independent of changes in systemic exposure.

Following validation of the mouse fluorophotometer with EIU, we next moved to the STZ-induced mouse diabetes model, to see if changes in vascular leakage related to chronic hyperglycaemia could be observed. After induction of diabetes, we first observed hyperglycaemia at 2 weeks in the mice (data not shown) that remained elevated until at least 10 weeks after STZ injection (Fig. 4a). Animals continued to gain weight in both non-diabetic control and STZ-diabetic mice, however at a slower rate in the STZ group, which did not reach significance (Fig. 4b).

Since fluorophotometry revealed low detection levels in the initial phases of measurements after fluorescein injection, the detection levels were less consistent and reliable. Therefore, we eliminated the early timepoints following injection, and included only measurements from 40 to 60 min. Levels of fluorescein in each eye compartment were significantly different from the non-diabetic control groups. In both the retina and anterior chamber (Figs. 4c and e), fluorescein levels were increased from 50 min in STZ-diabetic mice, relative to non-diabetic controls. In the vitreous, significant elevation of fluorescein was apparent at all time points measured (Fig. 4d). Plasma fluorescein was reduced in STZ mice (Fig. 4f), however values did not reach significance.

In a final validation experiment, we used the JR5558 mouse model of spontaneous choroidal vascularization (CNV). The JR5558 mouse has previously been used in preclinical studies for testing novel therapeutics^34,35^, so we wanted to use this model to monitor reductions in permeability longitudinally in the same animal eyes after VEGF neutralisation. Initially, we compared fluorescein accumulation in the JR5558 vs. age-matched C57Bl/6J control mice, to see how the presence of CNV impacted leakage. We used mice that were 13 weeks old for these experiments, which already had very well-established lesions. In the retina fluorescein increased between 45 and 55 min after subcutaneous administration of JR5558 mice (Fig. 5a), but was not significantly different from C57BL/6J at 60 min. In the vitreous, significant elevation of fluorescein was observed at time points from 45 to 60 min (Fig. 5b). For the anterior chamber, no significant changes were observed between C57Bl/6J and JR5558 mice (Fig. 5c), and plasma levels did not differ between mouse strains (Fig. 5d).

To examine the effect of VEGF neutralization on CNV leakage in the JR5558 mice, we administered IgG control, or anti-VEGF antibodies intraperitoneally twice to 8-week-old JR5558 mice. Fluorescein accumulation was measured in the vitreous only as retinal fluorescein levels were too variable (due to the presence of leaking neovascular lesions that created punctate areas of very high fluorescein signal), and the neovascular pathology is absent in the anterior chamber. Vitreous fluorescein levels were not significantly different in IgG and anti-VEGF groups at baseline. Following treatment, fluorescein levels in the vitreous did not change in the IgG group, however in the anti-VEGF group there was a significant reduction by 25% from baseline (*P *< 0.05; Fig. 5e,g). When we calculated each individual animal’s percentage change from baseline, similar effects were seen, with an overall 19% reduction in the anti-VEGF group, vs. a 7% increase in IgG control groups (Fig. 5f). Taken together, these data show that the fluorophotometer can be used in mice to provide longitudinal assessments of changes in permeability in response to treatment.

## Discussion

Here we demonstrate a method for real-time measurements of changes in vascular permeability, in vivo, in the mouse eye. The method was applied to mouse models of diabetic retinopathy, uveitis and choroidal neovascularisation, where increases in vascular leakage were observed when compared to control animals. In a final experiment, we demonstrated that it is possible to attenuate the elevated fluorescein levels using anti-VEGF treatment, and show that both pre- and post-treatment permeability changes can be measured within the same animal eyes.

As with any method, success can be determined by the optimum use of the technology. With the mouse fluorophotometer a number of factors have to be considered. It is essential to have accurate alignment of the optical head of the fluorophotometer with the centre of the mouse visual axis, to allow consistent scanning for reproducible data. As the machine is an optical device, based on focused excitation and detection of emission of light, it is important to have a visual axis as free from interference as possible. An initial problem was that under anaesthesia, even with regular lubrication, mouse eyes are prone to develop cataracts^36^, which makes recording of fluorescein levels in the posterior ocular tissues impossible. We found the solution was to apply contact lenses to the mouse eye following induction of anaesthesia, which prevented cataract formation. Furthermore, the contact lenses need to be free of residue left by fluid (e.g. PBS) drying on the surface of the lens. This can be resolved by rinsing the lenses in ultrapure water prior to use.

Also related to keeping a clear visual axis, the method can be sensitive to damage to the eye tissues, for example from intravitreal injections, or from agents that cause inflammation. We found that in our experiments using intravitreal LPS administration, we needed to titrate the dose of LPS, and perform intravitreal injections using 34 gauge blunt needles so that the incidence of vitreous haemorrhage or inflammatory cells in the anterior chamber was minimised. Variability between different batches of LPS was observed, so it was important to use the identical batch numbers where possible. Consistent eye dilation was also imperative; dilating agents were added after anaesthesia, with the same volume of Tropicamide administered to each eye.

In our experiments we tested fluorescein as a dye, since it is widely used in imaging and permeability studies, as well as having a favourable safety profile for repeated use in mice. Other dyes with different molecular weights may be better in fluorophotometry experiments for detecting leakage-dependent changes, but are beyond the scope of the current manuscript. We primarily used the subcutaneous route of administration for fluorescein, as it turned out to be most simple to perform, leading to fewer dosing errors, and reducing the signal to noise in comparison to the intravenous route. Another critical variable was the timing of the fluorescein dosing of the animals. We found that dosing the animals once they had been anaesthetised drastically decreased the variability of our results, perhaps since mice under anaesthesia are static and may have similar metabolic rates.

Mouse fluorophotometry has key advantages over some existing techniques to measure permeability, such as Evan’s blue, or other perfusion-based methods. Firstly, it is not necessary to sacrifice the animal in order to carry out the assay, meaning that animals can be potentially used for multiple experiments. This will help the practice of the 3Rs (reduction, refinement and replacement) in animal research^29^. Secondly, it is possible to monitor the vascular leakage in the same animal in a longitudinal manner using repeated measures, thus enabling researchers to calculate pre- and post-treatment effects, or development of permeability over time in ocular disease models. Third, the method can also be used in combination with more established methods for permeability, like Evan’s blue, and coupled with imaging techniques such as fluorescein angiography. Additionally, since the device is adapted for mouse use, this means that it’s possible to combine leakage measurements with targeted genetic manipulation in the species, to look for factors contributing to blood-retinal barrier breakdown. One downside though is that due to the significant differences in ocular and systemic anatomy and physiology, the results in mice should be taken with caution if trying to directly compare absolute leakage values across the species, including in human studies.

In summary we would recommend that the mouse fluorophotometer could be used in experiments where a clear visual axis can be maintained. Treatments or genetic alterations that lead for example to cataracts, hyphema or vitreous haemorrage may be better served by methods such as Evan’s Blue or other perfusion-based methods. However, if the visual axis can remain clear, then mouse fluorophotometry can be used over multiple time points alongside existing methods and can be used in conjunction with imaging-based methods like fluorescein angiography, where an ocular image may be informative. Furthermore, if the researcher wishes to compare their data with perfusion-based methods, they are still able to do so, if required.

In conclusion, we see the mouse fluorophotometer as a very useful tool for both basic research and drug discovery. As the next generation of therapeutics for wet age-related macular degeneration and diabetic macular oedema emerge, it is clear that having the best tools will help to identify drugs that interact with novel pathways, providing the optimum combination of fluid control and durability of treatment effect. The mouse fluorophotometer may offer an additional method to help separate out the best novel treatments.

## Materials and methods

### Animals

Table 1 shows information regarding strain, age, sex and supplier for the animals used in the different experiments throughout the study.

**Table 1:** Information regarding strain, age, sex and supplier for the animals used in the different experiments throughout the study.

Model/experiment	Strain	Supplier	Age at start of experiment	Sex
70 KDa Fluorescein, FITC-dextran IVT/dose response	C57Bl/6J	Charles River laboratories (Germany)	10–12 weeks old	Female
Endotoxin-induced uveitis (LPS IVT)	C57Bl/6J	Charles River laboratories (Germany)	12 weeks old	Female
STZ diabetes	C57Bl/6J	Charles River laboratories (France)	10 weeks old	Male
Spontaneous CNV/anti-VEGF	JR5558	Charles River laboratories outsourcing (Germany)	8 and 13 weeks old	Male and female

*FITC *= Fluorescein isothiocyanate, *IVT *= Intravitreal, *CNV* = Choroidal Neovascularisation, *LPS *= lipopolysaccharide, *STZ *= streptozotocin, *VEGF* = Vascular Endothelial Growth factor.

All animals received food and water *ad libitum*, in a 12-h day/night cycle, temperature-controlled environment. Animal experiments were approved by the Federal Food Safety and Veterinary Office of Switzerland (references BS-2730 and BS-2734) and conducted in strict adherence to the Swiss federal ordinance on animal protection and welfare, as well as according to the rules of the Association for Research in Vision and Ophthalmology Statement for the Use of Animals in Ophthalmic and Vision Research guidelines, and in compliance with the ARRIVE guidelines.

For intravitreal (IVT) lipopolysaccharide (LPS) injections, animals were anaesthetized using isoflurane 3%, O_2_ 100%. For all other experiments mice were anaesthetized using the subcutaneous combination of medetomidine (Dorbene®, 0.5 mg/kg, Graeub AG, Bern, Switzerland), midazolam (5 mg/kg, Roche Pharma AG, Grenzach-Whylen, Germany) and fentanyl (Curamed®, 0.05 mg/kg, Actavis Switzerland AG, Regensdorf, Switzerland). For recovery experiments, mice were then antagonized with a combination of buprenorphin (Bupaq®, 0.2 mg/kg, Streuli Pharma AG, Uznach, Switzerland), atipemazol (Alzane®, 2.5 mg/kg, Graeub AG, Bern, Switzerland) and flumazenil (Anexate®, 0.5 mg/kg, Roche Pharma AG, Grenzach-Whylen, Germany).

### Endotoxin-induced uveitis

Prior to IVT application, a solution of LPS (Cat L6529; Sigma, St Gallen, Switzerland) was prepared in sterile Dulbecco’s Phosphate Buffered Saline (DPBS; Life technologies, Switzerland) from frozen aliquots stored at − 20 °C with a final concentration of 1 ng/1 µl. Animals were anesthetized with isoflurane, and drops of local anesthetic (Novesin®; OmniVision, Switzerland) and mydriatic (Tropicamide 0.5% SDU Faure, Théa Pharma S.A., Schaffhausen, Switzerland)) applied. The eyes were rinsed with povidone iodine 5% (iso-Betadine® 5%, MEDA-Pharma GmbH & Co.KG, Bad Hombourg, Germany), ), followed by sterile PBS, then with a Nanofil Syringe and connected 34 gauge needle (NF34BL-2) (both World Precision Instruments International, Friedberg, Germany) 1 µl/eye LPS or PBS (control) was injected. Finally, animals were placed back into the housing box for recovery.

After 24 h, mice were anaesthetized using subcutaneous anaesthesia, then fluorescein angiography (FA), and ocular coherence tomography (OCT) images of the retina and vitreous were taken to confirm the presence of infiltrating inflammatory cells. Following imaging, mice underwent fluorophotometry.

### STZ diabetes

Animals were divided in two groups, receiving either streptozotocin (STZ; 50 mg/kg body weight, 7.5 mg/ml, prepared in 0.05 M sodium citrate buffer, pH 4.5) or PBS injected intraperitoneally (IP) for five consecutive days. STZ was prepared and stored in a refrigerator at least 30 min before administration. Five days after the last STZ or PBS doses, blood glucose was measured via tail vein puncture using an AlphaTrak blood glucose monitoring system (Abbott Laboratories Inc USA, Alameda,CA) and test strips (Alpha Trak2, Zoetis Schweiz, Zürich Switzerland). Development of diabetes was defined as a blood glucose levels higher group than 14 mmol/L (250 mg/dl). Animals with glucose levels lower than 250 mg/dl were not reinjected or included in the study. The animal’s weight and glucose were monitored throughout the study, and only mice with continuously elevated blood glucose levels were considered as diabetic. For fluorophotometry measurements, animals were measured at 10 weeks post-STZ.

### JR5558 spontaneous CNV mice

Male and female JR5558 mice^33^ were used throughout the studies. For the time course and anti-VEGF pharmacological treatment studies, animals were 13, and 8 weeks old, respectively. For drug treatment experiments, mice underwent baseline fluorescein angiography (FA)^34^ and fluorophotometry assessment on week 8 on the day prior to antibody dosing, to assign animals to treatment groups with statistically equal (*P *> 0.05; unpaired t-test) numbers of lesions and fluorescein concentrations in different eye compartments. Anti-VEGF (B20-4.1^37^) or IgG control antibodies were given I.P. one day after baseline, and then a repeat dose 3 days later (1 ml/100 g body weight, 2 doses in total). FA and fluorophotometry data were collected again one week after the initial antibody dose, to compare pre- and post-treatment effects of anti-VEGF and IgG control. For baseline and post-treatment assessment of JR5558 mice, the 45-min time point was selected for fluorophotometry.

### Fluorophotometry

Fluorophotometry measurements were carried out on a scanning ocular fluorophotometer (Fluorotron Master Research Mouse Edition; OcuMetrics, Inc., Mountain View, CA). A more detailed protocol describing the methods used in this manuscript can be found in the supplementary methods section.

**Figure 6:**
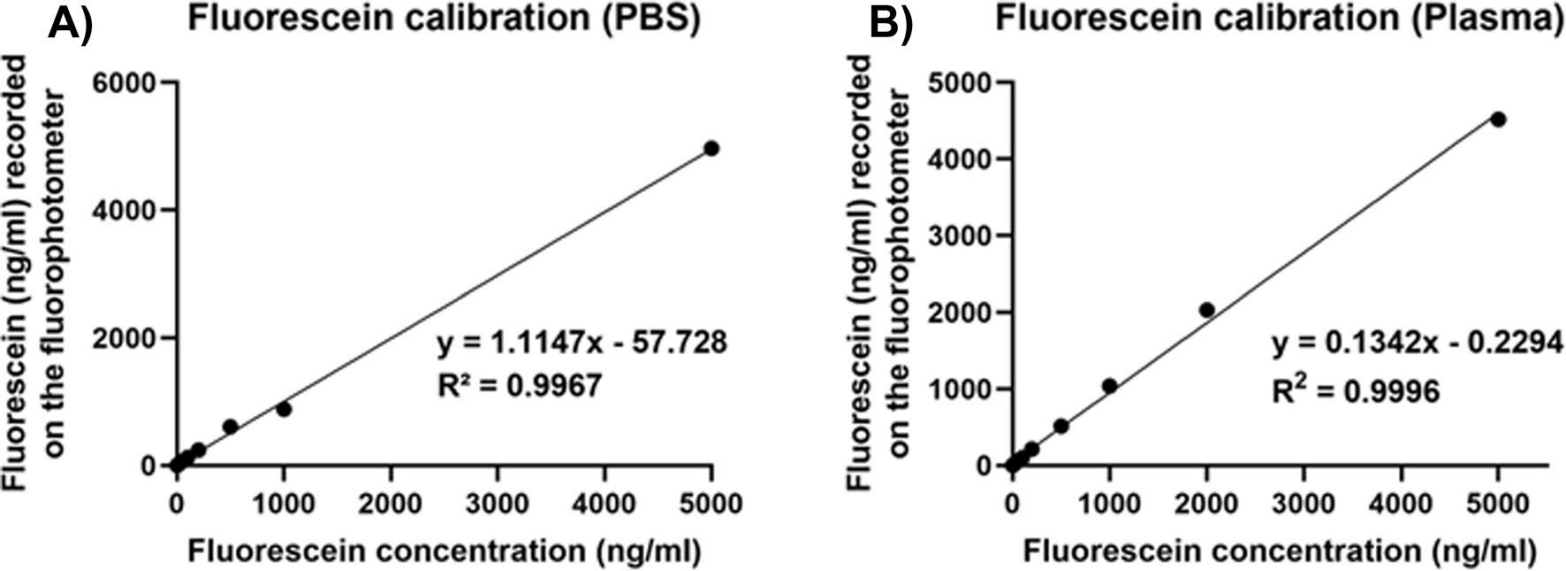
Fluorescein standard curves. Standard curves for fluorescein in (**A**) PBS and (**B**) plasma. Fluorescein standards were prepared in PBS or mouse plasma, then diluted further in PBS, before fluorescein concentration (ng/ml) was measured in a microcuvette. Regression analysis was carried out, with statistics shown on the graphs in the Figure.

To ensure that fluorescein concentrations measured in our experiments were in the linear range, we ran a fluorescein standard curve. Standard curves were not used for calculation of fluorescein levels in later experiments, since the fluorophotometer provides direct measurements of fluorescein in ng/ml. The standard curve was performed in microcuvettes with PBS as a diluent, using the microcuvette holder supplied by the manufacturer. Using this standard curve, a linear relationship was observed as the concentration of fluorescein increased (R^2^ > 0.99; Fig. 6), indicating that changes in fluorescein levels observed in our experiments were due to concentration-dependent differences.

For in vivo experiments, animals were anaesthetized using subcutaneous anaesthesia. Following anaesthesia eyes were dilated with 0.5% tropicamide (Tropicamide 0.5% SDU Faure, Théa Pharma, Schaffhausen, Switzerland), then 5 min later fluorescein solution (cat 46,960; Sigma-Aldrich, St Gallen, Switzerland) or FITC-dextran 70 kDa (cat 46,945; Sigma-Aldrich, St Gallen, Switzerland) was administered either IV, IVT or SC, at doses described in the results sections. For most consistent results, we observed it was best to administer fluorescein following anaesthesia.

After fluorescein injection, 3.2-mm plano contact lenses (Cantor and Nissel, Northamptonshire, UK) were placed on the mouse eyes. Contact lenses are used to prevent drying, which can lead to cataract formation. The animals were then placed on a temperature controlled (37 °C) stage, and the eyes aligned parallel to the optic device. Eye-scans were recorded from both left and right eyes using 450–490 nm excitation and 520–600 nm emission detection, at time points indicated in each result section. Eye scans took approximately 20 s per scan.

In some cases following fluorophotometry, blood samples (25 µl) were taken from the tail vein for plasma preparation (Microvette CB 300K2E; Sarstedt AG, Germany) to normalize scans to circulating fluorescein, to account for potential administration differences. Blood samples were centrifuged at 10,000 × g for 10 min, then 10 µl of the resulting plasma was diluted in 990 µl PBS in a microcuvette protected from light (PS Micro Photometer Cuvette 2 ml; LP Italiana, Milan, Italy) and scanned on the Fluorotron machine using the provided cuvette holder, also using 450–490 nm excitation and 520–600 nm emission detection.

To quantify fluorophotometry measurements, raw data was exported as txt. files, then plotted using Microsoft Excel. The average area under the curve (AUC) was calculated for 5 steps in each of the regions of the scans corresponding to retina, vitreous and anterior chamber peaks, as determined by eye compartment experiments (Fig. 1). Data not normalised to plasma fluorescein are expressed as fluorescein ng/ml ± SEM.

For plasma normalisation, circulating fluorescein was calculated by averaging the maximum peak values of duplicate scans. Fluorescein levels in eye compartments were then expressed as a ratio to plasma fluorescein, by dividing raw AUC averages by plasma values. Data are expressed as mean ± SEM ratio of ocular compartment:plasma fluorescein ng/ml.

### Statistics

Raw data were exported from the fluorophotometer in .txt files and copied into Microsoft Excel (Microsoft Corporation, Redmond, WA). Processing and analysis were carried out using Excel and GraphPad Prism 6 (GraphPad Software, La Jolla, CA) software. For statistical comparison of only two groups, an unpaired t-test was employed. One-way ANOVA followed by Newman Keul’s multiple comparisons test was used for comparisons of 3 or more groups. For time course data, Two-way Analysis of Variance ANOVA (alpha 0.05) with repeated measures, followed by multiple comparison tests were used (see Figure legends for further details). Outliers were removed prior to statistical analysis using Chauvenet's criteria, with outliers defined as being ± 2 × standard deviations of mean. Data are presented as Mean ± SEM with *P < 0.05, **P < 0.01, ***P < 0.001, and ****P < 0.0001.

## Supplementary Information

Supplementary Information.

## Data Availability

The datasets generated during and/or analysed during the current study are available from the corresponding author on reasonable request.
