# Supplementary material for: Real-time measurements of vascular permeability in the mouse eye using vitreous fluorophotometry
Source: Sci Rep. 2023 Jun 7;13:9226. doi: 10.1038/s41598-023-36202-4 (PMC10247817; doi:10.1038/s41598-023-36202-4)
Supplement: Supplementary file 1 — Supplementary Information. [file 41598_2023_36202_MOESM1_ESM.pdf]

Real-time measurements of vascular permeability in the mouse eye using vitreous fluorophotometry

Author list: Nadine Colé, Janina Thoele, Christoph Ullmer, Richard Foxton

Supplementary Methods - protocol for conducting an experiment using the mouse vitreous fluorophotometer

The following protocol describes the methods and steps required to carry out an experiment to measure the concentration of fluorescein in different compartments of the mouse eye over a period of 60 minutes, following subcutaneous administration of 50 mg/kg fluorescein. The method below describes a time course performed in a single animal, which has been used for the mouse strains and animal models described throughout the main manuscript. However the method can potentially be adapted for other mouse strains, and comparisons of treatment effects in different disease models using a single time point.

Materials

- FM-2 Fluorotron Master Laboratory Mouse Edition; OcuMetrics, Inc., Mountain View, CA. The fluorophotometer is supplied with a PC with the Fluorotron Master Software ready installed.
- Mice: strain and N depending on the experimental design
- Tropicamide 0.5%: SDU Faure, Théa Pharma, Schaffhausen, Switzerland
- Subcutaneous anaesthesia combination (prepare up to 7 hours in advance): medetomidine (Dorbene®, 0.5 mg/kg, Graeub AG, Bern, Switzerland), midazolam (5 mg/kg, Roche Pharma AG, Grenzach-Whylen, Germany) and fentanyl (Curamed®, 0.05 mg/kg, Actavis Switzerland AG, Regensdorf, Switzerland). The solution used was prepared at a volume of injection of 0.1 ml/10 g body weight.
- Subcutaneous anaesthesia reversal combination (prepare less than 24 hours in advance): buprenorphin (Bupaq®, 0.2 mg/kg, Streuli Pharma AG, Uznach, Switzerland), atipemazol (Alzane®, 2.5 mg/kg, Graeub AG, Bern, Switzerland) and flumazenil (Anexate®, 0.5 mg/kg, Roche Pharma AG, Grenzach-Whylen, Germany). The solution used was prepared at a volume of injection of 0.1 ml/10 g body weight.
- Heating pad covered with absorbent lab paper to keep animals warm during anaesthesia, when they are not placed on the fluorophotometer heated stage
- Fluorescein solution (prepare fresh for each experiment): Fluorescein sodium (cat 46960 Sigma-Aldrich, St Gallen, Switzerland) solution 1% (10 mg/ml) in phosphate buffered saline (Life technologies, Switzerland). Note: *the authors have also investigated Fluorescein 10% Faure solution (Fluorescein natricum 0.5 g/5 ml, Curatis AG, Leistal, Switzerland) with very similar results.*

- Capillary blood collection tubes for blood sampling: Microvette® CB 300K2E; Sarstedt AG, Germany)
- Cuvettes: PS Micro Photometer Cuvette 2ml; LP Italiana, Milan, Italy
- Mouse contact lenses: 3.2-mm plano contact lenses (Cantor and Nissel, Northamptonshire, UK). Note: *whilst the authors used this brand of contact lenses, others are available from other manufacturers, however the authors did not test these.*
- For cleaning contact lenses: 70% ethanol solution in water, and ultrapure water. Note: *clean the contact lenses before experiments by submerging in 70% ethanol solution and agitating with a Q-tip, before rinsing at least 3 times with ultrapure water.*
- Viscotears gel: Bausch and Lomb, Switzerland
- Ophthalmoscope: Heine Mini 3000 LED, Germany
- Dulbecco's Phosphate Buffered Saline (DPBS): REF 14190-094, Life technologies, Switzerland
- Stopwatch
- Centrifuge: Eppendorf 5427R, Switzerland

Methods

First prepare the fluorophotometer by switching the device and computer on, and opening the Fluorotron Master software. This will allow the stage to heat up to 37°C to keep the animal warm once measurements are ready to be taken. Enter the details of the mouse ID (or multiple IDs if running a larger experiment) into the software in advance to avoid time loss during a busy experiment. Also label capillary blood collection tubes for blood collection in advance, and also label cuvettes for plasma fluorescein measurement later in the experiment.

Next, prepare the animal for the experiment. Weigh the mouse and inject anaesthesia subcutaneously 0.1 ml per 10 g body weight into the loose skin around the neck. Anaesthesia is necessary to immobilise the animal and allow proper alignment of the eye for scans. Please be aware that the choice of anaesthesia can influence the measurements obtained from the fluorophotometer[1]. Once the animal is under anaesthesia, place it on a heating pad and dilate the eyes with 50 μl 0.5% Tropicamide. After 5 minutes check the eye is dilated using an ophthalmoscope, then add a drop of Viscotears gel, and gently place and push down a contact lens on top of the gel. *Note: contact lenses are essential to prevent cataract formation. Avoid air bubbles between the gel and the contact lens, and check that no eyelashes are trapped underneath the contact lens that might obstruct the visual axis*. Check that the pupils are fully dilated, and that the mouse is fully anaethetised before moving it to the fluorophotometer to begin measurements.

Place the mouse onto the heated stage at 37₀C, secure the head in place using the mouth bar, and fix the body in place using the velcro straps, to prevent loss of the position of the eye during the experiment. Then, using fine adjustment wheels on the stage, along with the viewfinder, centre the eye so that the beam will pass through the middle of the iris. Note: *some practice and repetition may be necessary to ensure that the placement of the eye results in the optimal signal*

measurement, when running an experiment with multiple animals. Furthermore, mouse hair or whiskers should be prevented from obstructing the visual axis. Once in position, inject the mouse with fluorescein and start the timer. Note: *in early validation work, the authors observed considerable variation when mice were injected with fluorescein before anaesthesia. Therefore it is recommended that measurements are only performed once fluorescein is injected after anaesthesia. It is also possible to inject fluorescein after anaesthesia, but before placing the animal on the stage if required, e.g for longer time points.* At the desired time points after fluorescein injection, switch off any lights in the room, or cover the mouse and fluorophotometer to prevent light interference, and perform scans. In between scans, lights can be switched on and measurements should be saved with appropriate file names ready to export during or after the experiment. Files can be exported as .txt files and the raw values copied into the data processing program of choice. For the experiments in this manuscript the authors used Microsoft Excel, as well as GraphPad Prism for data processing and statistical analysis, respectively.

For quantification of the different eye compartment measurements, it may be necessary to perform experiments similar to those described in Figure 2 of the manuscript. After identification of the eye compartments, the authors calculated the average area under the curve (AUC) for 5 steps in each of the regions of the scans corresponding to retina, vitreous and anterior chamber peaks, as determined by eye compartment experiments (Figure 2). Data that are not normalised to plasma fluorescein are expressed as mean fluorescein ng/ml ± SEM

Optionally following fluorophotometry, if required a blood sample (25 µl) may be taken for plasma preparation (Sarstedt AG, Germany, Microvette CB 300K2E, REF 16.444) in order to correct for fluorescein in the circulation. Plasma is obtained from the blood sample by centrifuging at 10,000 x g for 10 minutes, then 10 µl of the sample is diluted in 990 µl DPBS in a microcuvette (PS Micro Photometer Cuvette 2ml, 10x10x45mm REF112117, LP Italiana, Milan, Italy) and run on the fluorotron after removing the lens, and using the supplied cuvette holder also using 450-490 nm excitation and 520-600 nm emission detection, to quantify the amount of fluorescein in the plasma. Each plasma sample is run twice to obtain an average to be used for normalizing the ocular measurements to the amount of fluorescein circulating in the blood. For plasma normalisation, circulating fluorescein was calculated by averaging the maximum peak values of duplicate scans. Fluorescein levels in eye compartments were then expressed as a ratio to plasma fluorescein, by dividing raw AUC averages by plasma values. Data are expressed as mean ± SEM ratio of ocular compartment:plasma fluorescein ng/ml.

Finally, at the end of the experiment the mouse may be reversed from anaesthesia using the subcutaneous anaesthesia reversal combination described above, and after recovery, further experiments may be performed at later dates.

References

1. Toris, C.B., Fan, S., Johnson, T.V., Camras, L.J., Cassandra, L., Hays, C.L., Liu, H., Ishimoto, B.M. Aqueous Flow Measured by Fluorophotometry in the Mouse. *Invest Ophth Vis Sci* **57** (2016).

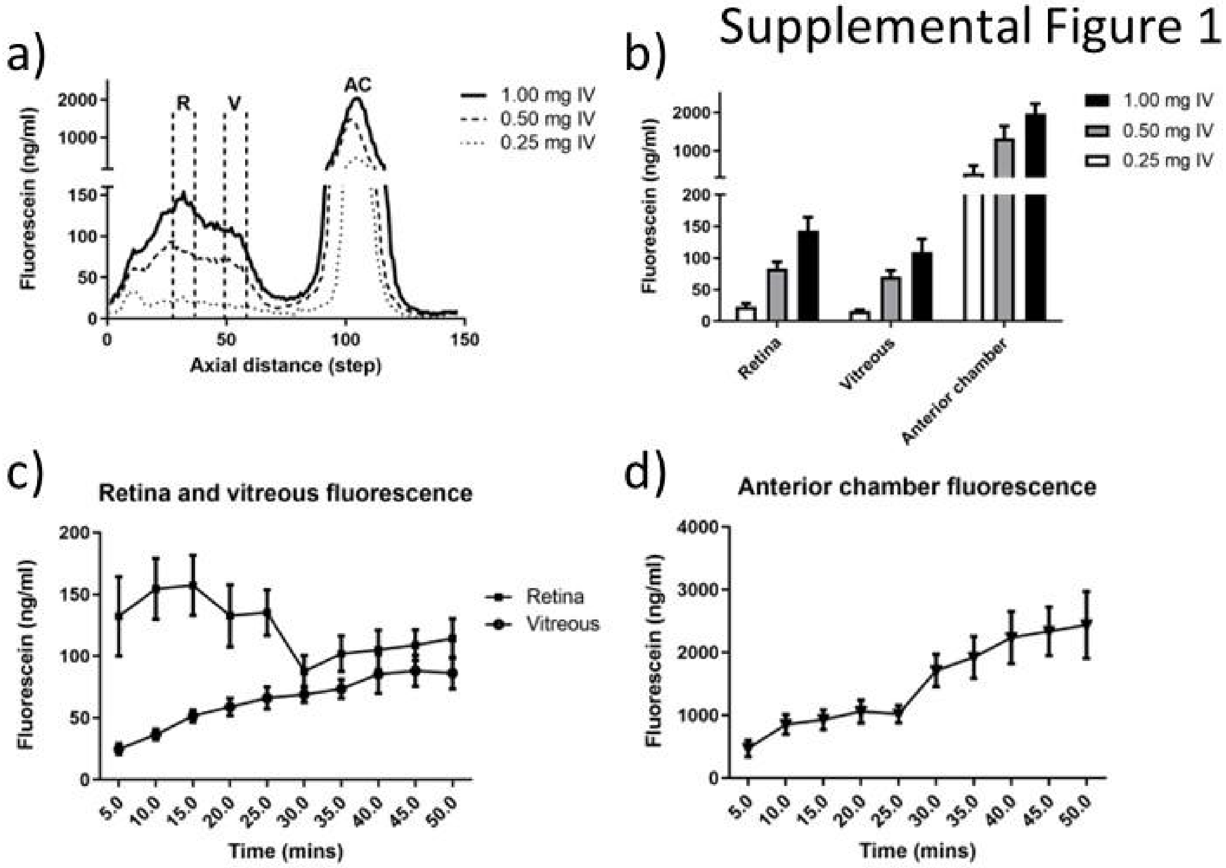

Supplemental Figure 1. Dose-response and time courses following intravenous administration of fluorescein to female C57Bl/6J mice. A) Representative scans and B) quantification of fluorescein in the retina, vitreous and anterior chamber averaged after 30 minutes, from mice injected with 0.25, 0.50 and 1.0 mg of fluorescein intravenously.

Time courses showing fluorescence levels in C) the retina and vitreous, and D) the anterior chamber of C57Bl/6J mice, 0-50 minutes after intravenous injection of 1 mg of fluorescein.

N = 4 - 8 mice for dose-responses in A) and B), and N = 8 - 11 mice for time courses in C) and D).
